# Diversity-sensitive palliative and hospice care in Germany – awareness, attitudes, and measures taken by service providers

**DOI:** 10.1186/s12904-025-01980-3

**Published:** 2026-01-08

**Authors:** Fabian Erdsiek, Munzir Mohamed Idris, Yüce Yilmaz-Aslan, Patrick Brzoska

**Affiliations:** Faculty of Health, School of Medicine, Health Services Research Unit, Witten/Herdecke University, Witten, Germany

**Keywords:** Diversity, Palliative and hospice care, Cross-sectional, Health services research

## Abstract

**Background:**

Studies have shown that needs and expectations concerning palliative and hospice care differ in relation to patients' diversity characteristics. Patients from minority groups may encounter various barriers that can prevent them from using or receiving appropriate, high-quality care. While corresponding measures and approaches exist that aim to address the diversity of patients, it is currently unclear, to what extent they are utilized by German palliative and hospice care facilities.

**Methods:**

To examine the current state of sensitivity or responsiveness to diversity of German palliative and hospice care providers, a mixed-mode cross-sectional survey was conducted using a random sample (*n* = 1901) of German hospices, palliative care units in hospitals, outpatient hospice services, specialized palliative care teams (so-called ‘SAPV teams’), and other service providers listed in the online guide of hospice and palliative care services of the German Association for Palliative Medicine (DGP).

**Results:**

Among the respondents (*n* = 346, response rate = 18.2%), the majority perceived diversity responsiveness as a development that is gaining importance but did not necessarily consider addressing it in their facility. While a majority of facilities (56.9%) considered diversity in their mission statement, dedicated activities to promote diversity responsiveness – like diversity trainings for staff members (35.3%), designated diversity commissioners (4.6%), or dedicated internal working groups (4.3%) – were less common. Similarly, written materials and services depending strongly on communication were not generally available. Barriers to implementing such services were mostly related to organizational difficulties (31.8%), missing knowledge on how to implement corresponding measures (28.3%), and decision makers not being convinced of the necessity (25.7%). Furthermore, 25.1% of respondents reported lacking financial resources.

**Conclusions:**

The surveyed palliative and hospice care facilities often did not address patient diversity sufficiently. Although a larger share of the respondents was generally aware of the need for diversity-sensitive care to improve patient orientation, corresponding structures and services were not commonly available. To address existing barriers, efforts to further raise awareness, as well as support measures to help facilities implement diversity-sensitive concepts are necessary.

**Supplementary Information:**

The online version contains supplementary material available at 10.1186/s12904-025-01980-3.

## Background

The population in Germany is increasingly diverse. Different dimensions of diversity have been consistently associated with varying needs and expectations concerning health services and their utilization [1–4]. Studies have shown differences in needs and expectations in palliative care services for different age groups, genders, sexual orientations, and cultural backgrounds, to only name some of the most referenced dimensions of diversity. For example, international studies have found that women were more likely to prefer palliative care over late curative treatments and more often had orders in place to prevent resuscitation, while male patients with advanced cancer were more likely to receive non-beneficial intensive care near death, which was mostly attributed to gender differences in end-of-life discussions with the patients’ oncologists [5–7]. Studies addressing palliative and end-of-life care needs of lesbian, gay, bisexual, transgender, queer, and other non-heterosexual, or non-cisgender (LGBTQ+) adults suggest a higher need for social support from respective communities and non-traditional or non-biological families (‘families of choice’), and increased need for services that demonstrate safety and non-discrimination (e.g. through LGBTQ+-friendly symbols) [8].

Additionally, patients of minority groups often encounter barriers when accessing health services in general, but also specifically relating to palliative and hospice care services or specialized services for end-of-life care. For example, studies showed that heteronormative attitudes, lack of recognition of same-gender partners and families of choice, as well as fear or experience of discrimination, stigmatization and neglect pose important barriers to the utilization of and satisfaction with palliative and hospice care [9, 10]. Currently, there are ca. 24.9 million people with a migration background in Germany, meaning they themselves migrated to Germany or are children of immigrants [11]. While the vast majority of them (83%) rated their ability to speak German as “good” or “very good”, only around 55% of refugees had similar ratings [12]. Furthermore, studies among migrant patients in different European countries have repeatedly shown that a lack of recognition of cultural issues surrounding death and dying, disregard for spiritual needs of patients from minority religions, as well as language barriers and discrimination are important barriers to using professional palliative and hospice care services [13–15]. This is often exacerbated by the strong reliance on family members, friends and relatives among migrant populations that is not or insufficiently addressed in palliative or hospice care settings [16]. In Germany, palliative and hospice care is provided by a range of different types of facilities. Palliative care units and palliative care advisory teams deliver services in hospital settings, mostly providing medical care to patients until they can be discharged into their homes or long-term care facilities, while (inpatient) hospices and palliative outpatient hospice services provide hospice and end-of-life care using a more holistic approach [17]. In addition, there are specialized palliative care teams (SAPV teams), i.e., multiprofessional teams that provide palliative medical and care services through trained specialists, coordinated with other providers of care, social support, and consultations [17].

Findings from other countries show that diversity-sensitive or diversity-responsive approaches to care can contribute to higher quality of care, improved patient satisfaction, and better outcomes (e.g. better health-related quality of life) [18, 19]. However, research on palliative and hospice care providers’ awareness of existing approaches, the perceived necessity of addressing the needs of diverse populations in palliative and hospice care delivery, as well as the prevalence of diversity-sensitive services in Germany is insufficient. The aim of this study was therefore to provide an overview over how and to what extent service providers of palliative and hospice care in Germany address patient diversity and which barriers affect the implementation of corresponding measures.

## Methods

For our study, we conducted a mixed-mode survey addressing a random sample of *n* = 1901 out of a total of about 3000 hospices, palliative care units in hospitals, outpatient hospice services, SAPV teams, and other service providers in Germany catering to adult patients that were listed in the “Wegweiser Hospiz- und Palliativversorgung Deutschland” database, a guide to palliative and hospice care services provided by the German Association for Palliative Medicine (DGP).

We contacted the respective persons responsible in management or administration (depending on the type of facility). Although staff members directly involved in patient care, medical services or other tasks involving direct patient contact are more likely to have a deeper insight into how diversity is addressed in practice, management and administration representatives are more likely to have information concerning structural and organizational aspects, networking and coordination activities, as well as comprehensive strategies and concepts used to address diversity. The initial postal survey wave was carried out between August and September 2020, followed by a second wave using the LimeSurvey platform for an online survey between October and November 2020. Facilities that chose not to participate were asked to fill out a short non-response questionnaire to identify reasons for non-participation.

Topics of the survey were perceived importance of responsiveness to diversity in general and in the respective facility, diversity responsive organizational structures, and measures, services, and strategies to address patient diversity, including culturally sensitive menu selection, tailored information and education materials, and other therapeutic and non-therapeutic services. Additionally, providers were asked about potential or existing barriers to implementing diversity-sensitive measures in their facility. The initial questionnaire was developed based on prior research on diversity responsiveness of other facilities providing healthcare services [17, 18, 20, 21] and supplemented by other research in the field. The questionnaire was pre-tested for understandability by health research experts. To account for a potential lack of knowledge or awareness of the topic in general, we added our own simplified definition of sensitivity to diversity (*“’sensitivity to diversity’ or ‘diversity responsiveness’ refers to the consideration of the diversity or variety of healthcare personnel and patients related to characteristics like gender*,* age*,* cultural background*,* language or disability”*) and presented it to respondents in the introduction of the questionnaire to ensure comparability of the answers. Questions about factual aspects, e.g., structural aspects, barriers, services offered, measures, and strategies, were designed as single- or multiple-choice items, aside from language-related materials and services, where the pre-test suggested a potentially larger share of facilities currently planning to implement such materials or services. For these questions, three options (available, in planning, not available) were presented. For questions relating to the perceived importance of diversity sensitivity we used 4-point Likert-style items (fully agree, somewhat agree, somewhat disagree, fully disagree).

Since the main goal of our study was to present an initial overview of measures, strategies, and structures, all data were analyzed descriptively using Stata 15.

## Results

 Of the 1901 facilities approached, 297 participated in the initial postal survey and another 49 in the subsequent online survey (total response rate = 346/1901 = 18.2%). Additionally, 140 facilities participated in the non-response survey. According to the non-response analysis, the main reason for not participating was the perception that addressing diversity was unnecessary or did not concern the facility's patients (54.3%). Other reasons were lack of time to participate (29.3%) and the respondent's feeling that they were the wrong contact person (12.1%).

Most facilities participating were outpatient hospice services (50.3%), followed by inpatient facilities (hospices or palliative care units in hospitals) (31.8%) and SAPV teams (10.7%). Most of the facilities included were run by non-profit providers (69.6%), fewer facilities were operated by public (15.6%) or private providers (10.1%). On average, facilities had 55.3 employees, with answers ranging from 1 employee to 900 employees, and catered to on average of 443.6 patients per year (Table 1).

**Table 1:** Structural aspects and characteristics of participating facilities according to respondents (Survey of facilities providing hospice and palliative care services in Germany, 2020, *n* = 346; n.s.: not specified)

Structural aspects		*n*	%
Type of facility	In-patient service (hospice, palliative care unit)	110	31.8
	Outpatient hospice service	174	50.3
	SAPV team	37	10.7
	Other	24	6.9
	n.s.	1	0.3
Ownership	Public (e.g., communal, state-owned)	54	15.6
	Private not-for-profit (including churches)	242	69.9
	Private for-profit	35	10.1
	n.s.	15	4.3
		**Mean (n)**	**Min – max (n)**
Number of staff		55.3	1–900
Patients per year		443.6	8–15,000

Most respondents (93.1%) considered their facility to sufficiently address the needs of different patient groups. Fewer respondents (69.1%) generally agreed to the statement that a diversity-sensitive approach to care is necessary. While 82.9% of respondents considered diversity-sensitive care to become more important in the future for hospices and palliative care providers in general, only 42.8% reported planning for a more diversity-oriented approach. Education for staff members on diversity and diversity-sensitive care was generally seen as important by most respondents (70.2%), while 20.8% disagreed somewhat and 5.2% fully disagreed. While 34.7% of respondents fully agreed that practical recommendations were likely to improve implementation of diversity-sensitive measures, 51.5% somewhat agreed and altogether 8.7% generally disagreed (Table 2).

**Table 2:** Awareness and perceived importance of diversity sensitivity for palliative and hospice care facilities (Survey of facilities providing hospice and palliative care services in Germany, 2020, *n* = 346; n.s.: not specified)

	Fully agree	Somewhat agree	Somewhat disagree	Fully disagree	*n*.s.
Agreement to statement:	%	%	%	%	%
“Our facility addresses the needs of different patient groups.”	65.9	27.2	5.2	0.3	1.5
“A diversity-sensitive approach to care is necessary for our facility.”	27.2	41.9	21.1	4.3	5.5
“In the future, we are planning to have a more diversity-oriented approach to care.”	7.8	35.0	36.7	12.7	7.8
"A diversity-sensitive approach to care is going to become more important for many facilities in the future.”	30.6	52.3	11.6	1.2	4.3
“It is important that our staff receive education on how to address diversity.”	23.4	46.8	20.8	5.2	3.8
“Practical recommendations could improve the implementation of diversity-sensitive measures.”	34.7	51.5	7.2	1.5	5.2

The majority of respondents reported that their facility had included sensitivity to diversity in their mission statement (56.9%), while 13.6% reported planning to do so. In contrast, only 35.3% of facilities were offering regular training courses, seminars, or other forms of education for staff members to increase sensitivity to diversity, while 15.9% were planning on doing so. Similarly, 26.3% of the facilities were part of professional networks, working groups or other organizations focusing on diversity in general or on selected dimensions of diversity. While 11.3% were currently planning to do so, 59.5% did not participate in any type of professional organization addressing diversity. Only 4.6% of the respondents stated that their facility currently had a designated diversity commissioner or similar representative, while 2.6% were planning to implement such a position. According to the respondents, internal working groups on sensitivity to diversity were implemented in 4.3% of the facilities, while 4.9% were currently planning to implement them.

One way of addressing language barriers is to provide oral or written information in different languages. Since not all facilities offer any written materials or specific services, the following numbers are in relation to those facilities, that reported to generally offer the respective materials and services to their patients (Table 3). In this respect, 45.9% of respondents reported that their facilities currently offered forms of consent in languages other than German, while 52.1% offered other forms and information leaflets in languages other than German. In contrast, bi- or multilingual menus (16.2%) and homepages were less common (9.3%). According to the respondents, counseling services in other languages than German were offered by 54.9%, while anamnesis, admission and exit interviews in other languages were offered by 49.0% of the facilities. Less common were multilingual medical and physiotherapist treatments (36.8%) and psychological, occupational, and other forms of therapy relying strongly on communication (27.6%). The majority of respondents reported offering all of these materials and services only in German (57.8%). Of the facilities offering materials and services in other languages, 8.6% offered materials in three or more additional languages, 11.9% reported two additional languages and 66.5% reported one other language. All the aforementioned materials and services were most commonly offered in Turkish (34.4%), English (30.1%), or Russian (28.0%), while Arabic (19.7%) and Polish (12.7%) were less common. Only 4.9% of respondents reported other languages.

**Table 3:** Care-related materials and services offered in languages other than German in relation to all facilities (differing totals) offering these materials or services in general (Survey of hospices and palliative care services in Germany, 2020, *n* = 346)

Care-related materials and services	Available	In planning	Not available	Total (*n*)
Written materials in languages other than German				
Consent form	45.9%	8.6%	45.5%	209
Other forms and information leaflets	52.1%	9.6%	38.4%	219
Homepage/online presence	9.3%	12.2%	78.5%	205
Menus	16.2%	2.6%	81.2%	117
Services offered in languages other than German				
Anamnesis, admission, and/or exit interviews	49.0%	3.1%	47.9%	192
General counseling	54.9%	4.0%	41.2%	226
Medical and/or physiotherapist treatment	36.8%	5.8%	57.4%	155
Psychological, occupational, and other communication-heavy forms of therapy	27.6%	6.9%	65.5%	145

To address gender-specific and cultural needs, 37.5% of the facilities offered tailored information and education workshops or seminars (Table 4). 56.5% of the respondents stated that their facilities offered treatment/care exclusively by staff of the same sex if requested. Additionally, 39.4% reported offering individual consultations or group discussions for different religious groups. 70.3% of the facilities reported offering counseling through cooperating spiritual counselors from different religions. Among facilities that generally offered catering, 39.7% reported having a menu selection that was considerate of cultural or spiritual needs (e.g. kosher, helal, vegan food), with 43.8% providing the option to get meals outside usual service times (e.g. after sundown during Ramadan) (Table 5). Among the facilities, 57.1% offered accommodation for partners or family members. More than half of the facilities reported providing the option to decorate patient rooms according to patients’ cultural and spiritual needs, while 30.6% had neutrally decorated farewell rooms and 27.9% had neutrally decorated prayer rooms.

**Table 4:** Additional diversity-sensitive therapeutic services offered by all 216 facilities generally offering such services (Survey of facilities providing hospice and palliative care services in Germany, *n* = 346)

Additional therapeutic services	*n*	%
Tailored information and education workshops or seminars	81	37.5
Consultations (individual or group discussions) for different religious groups	85	39.4
Option to receive care exclusively from same-sex staff	122	56.5

**Table 5:** Additional diversity-sensitive non-therapeutic services offered by all 219 facilities generally offering such services (Survey of facilities providing hospice and palliative care services in Germany, *n* = 346)

Additional non-therapeutic services	*n*	%
Culturally sensitive menu selection (e.g. kosher, helal, vegan)	87	39.7
Meals available outside usual service times (e.g. after sundown during Ramadan)	96	43.8
Counseling through cooperating spiritual counselors from different religions	154	70.3
Accommodation for partners and/or family members	125	57.1
Option to decorate patient rooms according to patients’ cultural and spiritual needs	111	50.7
Neutrally decorated farewell rooms	67	30.6
Neutrally decorated prayer rooms	61	27.9

According to the respondents, the most common barriers to implementing diversity-sensitive measures were organizational difficulties (31.8%). In addition, 28.3% reported not knowing how to implement such measures. In terms of awareness, 25.7% of respondents stated that in their facility, not all executives or decision makers were convinced of the necessity of such measures, while 10.1% reported a lack of motivation among staff members. Furthermore, 25.1% reported a lack of financial resources, while 17.3% reported missing incentives from funding bodies. In contrast, 26.9% reported no barriers. According to the respondents, 14.2% of the facilities had no intention to introduce diversity-sensitive measures at all (Table 6).

**Table 6:** Barriers to implementing diversity-sensitive measures according to respondents (multiple response options possible) (Survey of hospices and palliative care services in Germany, *n* = 346)

Reported barriers	*n*	%
Organizational difficulties	110	31.8
No knowledge of how to implement diversity-sensitive measures	98	28.3
Not all responsible decision-makers are convinced of the necessity	89	25.7
Lack of motivation among staff members	35	10.1
Lack of financial resources	87	25.1
Missing incentives from funding bodies	60	17.3
No barriers	93	26.9
Not applicable, currently no intention to implement diversity-sensitive measures	49	14.2

## Discussion

Addressing the diversity of patients is an essential part of patient-centered care. This is specifically true for palliative and hospice care, where the experience of patients and the perceived quality of the patient-provider interaction are the most important outcomes, in contrast to curative or rehabilitative treatments. Our study shows that addressing diversity in care is considered an important topic by most facilities, although a certain proportion of respondents view it as a general trend that does not concern their own facilities. While most respondents agree that sensitivity to diversity is necessary and will become increasingly important in the future, most facilities currently address diversity only in a rudimentary manner. According to the respondents, the majority of facilities address diversity in their mission statement, but rarely implement corresponding structures, processes and/or services. While this might point to diversity responsiveness being used as a marketing tool rather than an actual guiding principle of care, it could also hint at underlying implementation barriers.

Only around one third of facilities offered regular trainings or workshops on diversity to their staff, and less than 5% had internal working groups or designated diversity representatives at the time of the study. This suggests a very limited understanding of how to address patient diversity adequately. Studies have shown that, specifically considering care services for LGBTQ+ patients, dedicated training and awareness measures are important to overcome heteronormativity, create a sense of safety, and adequately integrate chosen families and partners, as well as to prevent discrimination, isolation, and re-closeting [22–24]. Additionally, considering the increasing number of foreign nationals and Germans with a migration history, addressing cultural and spiritual needs of patients of different spiritual and religious beliefs comprehensively in all facilities becomes more important particularly in palliative and hospice care, where these aspects are of utmost relevance for patients [25, 26]. Studies show that patients from different cultural backgrounds often value certain aspects of palliative and end-of-life care differently, leading to a high potential of misunderstandings, which may affect the quality of care considerably [16, 27]. Specific trainings addressing these aspects could greatly improve patient satisfaction and reduce or prevent misunderstandings and conflicts [28, 29].

Furthermore, language barriers are likely to impair social interactions and can limit the quality of service delivery, which only some of the facilities in our study attempt to address [14, 27]. While consent forms and other general written information materials were often available in more than just German language, homepages and menus were mostly not available in languages other than German. Given that the internet has become a major source for health-related information [30], the large percentage of facilities without an online presence in a language other than German could be a barrier to accessing these services. In addition, treatments and therapies are only offered in different languages by a minority of facilities, whereas admission interviews, general counseling, and other non-specific verbal interactions are offered in different languages by around half of the facilities in our study. This further suggests that language barriers might strongly affect access to palliative and hospice care and specifically have a negative impact on receiving high-quality treatment. These factors could increase isolation, reduce access to social support, and affect patient satisfaction and psychological well-being.

Specific consideration for gender-related, spiritual or cultural needs in services offered to patients is limited. Services to address these needs are mostly offered when they do not require additional resources, e.g., through cooperations with spiritual counselors, as options to decorate patient rooms according to patients’ cultural and spiritual needs, or as part of more general measures to address needs of the majority population, e.g. accommodations for friends and relatives, or the option to be treated exclusively by same-sex staff. This suggests economic considerations as a potential hindrance to offering diversity-sensitive services. This is corroborated by our findings showing that a lack of financial resources and missing incentives from funding bodies are considered to be barriers to implementing such services or measures. Additionally, economic evaluations of palliative care models and concepts for culturally competent or otherwise diversity-sensitive care in general are scarce and their generalizability is often limited [31, 32]. This leaves open questions concerning their financial viability, potentially increasing hesitance among providers to implement such measures.

According to the respondents, organizational difficulties and lack of competence and knowledge on how to implement and sustain diversity-sensitive measures are among the most important barriers to diversity-sensitive care. Considering possible interactions of different dimensions of diversity (intersectionality) and the potentially exacerbated challenges for patients who are disadvantaged in more than one regard, e.g. homosexual immigrants, programs and interventions aimed at one dimension of diversity exclusively seem insufficient to provide adequate diversity-sensitive care [33, 34]. In this regard, palliative and hospice care facilities require additional support in choosing and implementing concepts and measures, e.g. through practice-oriented guidelines and handbooks, to effectively address patient diversity. Furthermore, efforts to incorporate diversity aspects in professional training and education of palliative care professionals could prove beneficial to improve patient-provider relationships and increase acceptance of corresponding measures among professionals [35]. Finally, awareness of the importance of diversity-sensitive care as an integral part of patient orientation and satisfaction with care is limited among many respondents, suggesting the need for further communication efforts, and dedicated public health policies to address these challenges.

## Strengths and limitations

To our knowledge, this is the first study presenting an overview of the current state of responsiveness to diversity in palliative and hospice care in Germany. Using a mixed-mode survey, we were able to reach facilities relying on traditional communication modes as well as facilities using electronic communication. Our survey addresses different aspects of responsiveness to diversity, ranging from awareness and attitudes to existing measures and structures, as well as barriers to implementation. This allows for an extensive overview over the current state of care as well as identifying potential focal points for efforts to improve diversity responsiveness and awareness. However, some limitations need to be addressed as well. Due to the small sample size, or more precisely the size of some subgroups of the sample, a more detailed, stratified analysis taking into account aspects like urbanity, type of facility/service or the size of the facility could not be performed. Furthermore, private not-for-profit providers are overrepresented in our sample, suggesting that our sample may not be fully representative of German palliative and hospice care facilities. Despite being comparable to other related surveys in the field [21], the low response rate and answers to our non-response survey further suggest that our sample may overly represent facilities already interested or aware of diversity as an important aspect to consider in healthcare. This could mean that awareness of the importance of addressing diversity as well as the prevalence of diversity-sensitive structures and measures are lower than our findings suggest. In the same respect, personal biases of the respondents could have influenced some of the answers and may have led to an overestimation of the acceptance of diversity and an underestimation of existing barriers. Availability of information about diversity-sensitive measures and services may have differed between respondents. To minimize this risk, we only addressed management or administration representatives for our survey. Furthermore, while we included questions on the availability of services and materials in languages other than German, we did not gather any information about the use of easy or simplified language. Accordingly, patients with limited literacy may not fully benefit from these materials and services.

## Conclusions

German palliative and hospice care facilities mostly do not address patient diversity sufficiently. While a larger proportion of facilities are aware of the necessity to be diversity-sensitive to improve patient-centered care, dedicated services and materials are not far-spread. Further efforts to raise awareness and provide more evidence for the necessity and benefits of diversity sensitivity are needed. This includes evaluation studies of respective measures and concepts, as well as increased science communication efforts. To address the lack of knowledge on how to implement diversity-sensitive measures and overcome organizational challenges, practice-oriented guidelines or handbooks taking into account potential facility-specific limitations and external conditions are important. In addition, financial concerns are an important barrier to many facilities. Furthermore, evaluation studies to assess the efficiency of existing measures and political efforts to allocate additional resources are necessary.

## Supplementary Information

Supplementary Material 1.

## Data Availability

The datasets used and/or analysed during the current study are available from the corresponding author on reasonable request.

## Abbreviations

LGBTQ+: Lesbian, gay, bisexual, transgender, queer, and other non-heterosexual or non-cisgender persons
SAPV team: Specialized multiprofessional outpatient palliative care team
DGP: German association for palliative medicine
