# Supplementary material for: Diversity-sensitive palliative and hospice care in Germany – awareness, attitudes, and measures taken by service providers
Source: BMC Palliat Care. 2026 Jan 8;25:11. doi: 10.1186/s12904-025-01980-3 (PMC12781678; doi:10.1186/s12904-025-01980-3)
Supplement: Supplementary file 1 — Supplementary Material 1. [file 12904_2025_1980_MOESM1_ESM.pdf]

Fragebogen

Diversitätssensible Versorgungsstrategien in der Palliativ- und Hospizversorgung

Sehr geehrte Damen und Herren,

mit diesem Fragebogen möchten wir diversitätssensible Versorgungsstrategien in der Palliativ- und Hospizversorgung ermitteln. Der Fragebogen kommt im Rahmen der Studie DiVerPH zum Einsatz. Nähere Informationen dazu finden Sie im beiliegenden Anschreiben.

Zwei Begriffe, die im Fragebogen verwendet werden, möchten wir vorab erläutern:

- **Diversitätssensibilität:** Mit Diversitätssensibilität oder diversitätssensiblem Umgang ist die Berücksichtigung der Vielfalt von Mitarbeitern/innen oder Patienten/innen im Hinblick auf Faktoren wie z.B. Geschlecht, Alter, Kultur, Sprache und Behinderung gemeint.

- **Maßnahmen diversitätssensibler Versorgung:** Hiermit sind Angebote, Leistungen und Maßnahmen gemeint, die zu einem diversitätssensiblen Umgang mit Mitarbeitern/innen und/oder Patienten/innen beitragen. Hierzu zählt zum Beispiel, mehrsprachige Informationen vorzuhalten oder eine durchgehende Barrierefreiheit zu gewährleisten. Weitere Beispiele finden Sie in diesem Fragebogen.

Im Fragebogen bitten wir Sie, die Antworten/Aspekte, die für Ihre Einrichtung zutreffend sind, anzukreuzen. Wir würden uns freuen, wenn Sie jede Frage beantworten würden. Mit „Einrichtung“ meinen wir dabei sowohl Palliativstationen und Hospize, als auch ambulante Dienste, Teams oder Praxen niedergelassener Palliativmediziner/innen. Wenn Sie sich an einigen Stellen nicht sicher sind, reichen uns auch Ihre Einschätzungen. Fragen, die nicht zu Ihrer Einrichtung passen, können Sie einfach auslassen. Das Ausfüllen des Fragebogens nimmt ca. 10-15 Minuten in Anspruch. Sollten Sie Fragen zur Studie oder zum Ausfüllen des Fragebogens haben, stehen wir Ihnen sehr gerne zur Verfügung.

Für Ihre Unterstützung danken wir Ihnen herzlichst.

Organisationsbezogene Merkmale

**Bitte geben Sie die Art der Trägerschaft Ihrer Einrichtung an:**		
☐ Staatlich/kommunal	☐ Freigemeinnützig	☐ Privat

**Bitte geben Sie an, wie viele Mitarbeiter/innen Ihre Einrichtung/Abteilung (Ehrenamtliche eingeschlossen) ungefähr hat:**	☐☐☐
**Bitte schätzen Sie, wie viele Menschen Ihre Einrichtung im Jahr ungefähr betreut/behandelt:**	☐☐☐☐☐

**Um was für eine Art der Einrichtung handelt es sich?**	
☐ Palliativstation / Hospiz	☐ Ambulanter Hospizdienst
☐ Spezialisierte Ambulante Palliativversorgung (SAPV-Team)	☐ Praxis mit Spezialisierung für Palliativmedizin
☐ Sonstige: ________________________________	

Unsere Einrichtung ist spezialisiert auf eine Zielgruppe (z.B. Kinder): ☐ nein ☐ ja, und zwar: ________________

Organisationsbezogene Maßnahmen

**Welche der folgenden Aspekte zur Diversitätssensibilität wurden in Ihrer Einrichtung umgesetzt bzw. sind in Planung?**	**Nein**	**In Planung**	**Bereits umgesetzt**
Berücksichtigung diversitätssensibler Aspekte im Leitbild	☐	☐	☐
Regelmäßige Fortbildungen/Schulungen/Fachberatungen für Mitarbeiter/innen zur Förderung der Diversitätssensibilität	☐	☐	☐
Benennung oder Einstellung von Diversitätsbeauftragten	☐	☐	☐
Gründung von Arbeitsgruppen zum Thema Diversitätssensibilität	☐	☐	☐
Einbeziehung externer Dienstleister oder Organisationen (z.B. Beratung/ Coaching) in die Planung oder Umsetzung diversitätssensibler Maßnahmen	☐	☐	☐
Beteiligung an Arbeitskreisen, Netzwerken o.Ä. zum Thema Diversität bzw. einzelnen Diversitätsmerkmalen	☐	☐	☐
Gezielte Öffentlichkeitsarbeit zum Thema Diversitätssensibilität	☐	☐	☐
Überprüfung des Erfolgs diversitätssensibler Maßnahmen	☐	☐	☐
Sonstige, und zwar: ____	☐	☐	☐

**Welche Dimensionen von Diversität werden in Ihrer Einrichtung berücksichtigt?**	**Gar nicht**	**Kaum**	**Mittel**	**Stark**	**Sehr stark**
Migrationshintergrund/Sprache	☐	☐	☐	☐	☐
Religion/Glaube	☐	☐	☐	☐	☐
Kultur	☐	☐	☐	☐	☐
Sexuelle Identität	☐	☐	☐	☐	☐

**Welche Dimensionen von Diversität werden in Ihrer Einrichtung berücksichtigt?** (fortgesetzt)	**Gar nicht**	**Kaum**	**Mittel**	**Stark**	**Sehr stark**
Geschlechterpräferenz	☐	☐	☐	☐	☐
Alter	☐	☐	☐	☐	☐
Körperliche oder geistige Einschränkungen	☐	☐	☐	☐	☐
Sonstige: ______________________________	☐	☐	☐	☐	☐

Umgang mit der Diversität von Mitarbeitern/innen

**Achten Sie bei der Personalpolitik darauf, dass Ihr Personal einen Mindestanteil hat an:** (Mehrfachnennung möglich)	
☐ Mitarbeiter/innen mit Migrationshintergrund	☐ Ältere Mitarbeiter/innen
☐ Mitarbeiter/innen mit körperlichen oder geistigen Einschränkungen	☐ Männliche bzw. weibliche Mitarbeiter/innen

**Achten Sie in der Personalpolitik auf die folgenden Aspekte:**<br>**(Bitte teilen Sie zu jeder Aussage mit, wie sehr sie zutrifft)**	**Trifft gar nicht zu**	**Trifft eher nicht zu**	**Trifft eher zu**	**Trifft voll zu**
Durch spezielle Beratungs- und Förderangebote erhöhen wir den Frauenanteil in Führungspositionen.	☐	☐	☐	☐
Durch Sprachkurse fördern wir Deutschkenntnisse unserer Mitarbeiter/innen.	☐	☐	☐	☐
Durch Sprachkurse fördern wir Fremdsprachenkenntnisse unserer Mitarbeiter/innen.	☐	☐	☐	☐
Wir werben gezielt Mitarbeiter/innen im Ausland an.	☐	☐	☐	☐
Wir achten darauf, dass unser Personal vielfältig in Bezug auf Alter, Geschlecht, Migrationshintergrund oder körperliche/ psychische Einschränkungen bleibt.	☐	☐	☐	☐
Wir schreiben vakante Stellen über verschiedene Quellen aus, um explizit benachteiligte Menschen (z.B. mit Behinderungen) anzusprechen.	☐	☐	☐	☐
Wir haben Maßnahmen (z.B. Leitbilder) implementiert, um Mitarbeiter/innen und Patienten/innen unterschiedlicher sexueller Identität (z.B. Transgender etc.) anzusprechen.	☐	☐	☐	☐
Wir haben Angebote (z.B. Mentorenprogramme) um neuen Mitarbeitern/innen mit besonderen Bedarfen (z.B. Behinderungen) den Einstieg zu erleichtern.	☐	☐	☐	☐
Sonstiges, und zwar: ______________________________	☐	☐	☐	☐

Umgang mit der Diversität von Patienten/innen

Information und Kommunikation

Im Folgenden geht es darum, inwiefern Angebote und Materialien in Ihrer Einrichtung auf diverse Patienten/innengruppen zugeschnitten sind. Sollten Sie die entsprechenden Angebote oder Materialien *gar nicht* anbieten, kreuzen Sie bitte „nicht zutreffend“ an.

**Werden die folgenden Materialien in verschiedenen Sprachen angeboten (sofern jeweils zutreffend)?**	**Nicht zutreffend**	**Nein**	**In Planung**	**Ja**
Schriftliche Einverständniserklärungen	☐	☐	☐	☐
Schriftliche Form- und Merkblätter	☐	☐	☐	☐
Internetseiten/Homepage	☐	☐	☐	☐
Speisepläne	☐	☐	☐	☐
Sonstige, und zwar: ______________________	☐	☐	☐	☐

Falls Materialien mehrsprachig angeboten werden, nennen Sie bitte die häufigsten Sprachen hiervon: (Mehrfachantworten möglich)

☐ Englisch ☐ Türkisch ☐ Russisch ☐ Polnisch ☐ Arabisch ☐ Sonstige: ______________

**Werden die folgenden Leistungen in Ihrer Einrichtung in verschiedenen Sprachen angeboten (sofern jeweils zutreffend)?**	**Nicht zutreffend**	**Nein**	**In Planung**	**Ja**
Anamnese-, Aufnahme- oder Entlassungsgespräche	☐	☐	☐	☐
Beratungs- oder Betreuungsgespräche	☐	☐	☐	☐
Medizinische und/oder physiotherapeutische Behandlungen	☐	☐	☐	☐
Psychologische, ergotherapeutische oder andere sprachlastige Therapien	☐	☐	☐	☐

Falls Leistungen mehrsprachig angeboten werden, bitte nennen Sie die häufigsten hiervon: (Mehrfachantworten möglich)

☐ Englisch ☐ Türkisch ☐ Russisch ☐ Polnisch ☐ Arabisch ☐ Sonstige: ______________

**Werden die folgenden Materialien/Formulare in Ihrer Einrichtung sprachunabhängig (Verwendung von Abbildungen, Piktogrammen) angeboten (sofern jeweils zutreffend)?**	**Nicht zutreffend**	**Nein**	**In Planung**	**Ja**
Informationsmaterial	☐	☐	☐	☐
Therapiepläne	☐	☐	☐	☐
Beschilderung oder Wegweiser	☐	☐	☐	☐
Speisepläne	☐	☐	☐	☐
Sonstige, und zwar: ______________________	☐	☐	☐	☐

**Inwieweit werden in Ihrer Einrichtung die folgenden Personen zum Dolmetschen/Übersetzen für Patienten/innen mit geringen Deutschkenntnissen hinzugezogen?**	**Nicht zutreffend**	**Nein**	**In Planung**	**Ja**
Angehörige, Freunde oder Bekannte der Patienten/innen	☐	☐	☐	☐
Andere Patienten/innen, die sich in der Einrichtung befinden	☐	☐	☐	☐
Ärztliches, pflegerisches oder therapeutisches Fachpersonal (und kooperierende Experten) mit gleicher Muttersprache	☐	☐	☐	☐
Sonstiges Personal (z.B. Ehrenamtliche) mit gleicher Muttersprache	☐	☐	☐	☐
Speziell ausgebildete professionelle Dolmetscher/innen	☐	☐	☐	☐
Kooperierende Seelsorger oder Mitglieder von Kulturgemeinden	☐	☐	☐	☐
Sonstige, und zwar: ________________________________	☐	☐	☐	☐

Unterbringung, Versorgung und Verpflegung

Im Folgenden geht es darum, inwiefern therapeutische und nicht-therapeutische Angebote in Ihrer Einrichtung auf diverse Patienten/innengruppen zugeschnitten sind. Sollten die entsprechenden Leistungen aufgrund der Art Ihrer Einrichtung (z.B. ambulante Dienste*) gar nicht* zur Verfügung stehen, kreuzen Sie bitte „Die genannten Angebote treffen bei unserer Einrichtungsart nicht zu“ an.

**Welche der folgenden therapeutischen bzw. Betreuungs- und Begleitungsangebote werden (ggf. unter Einbeziehung externer Partner, wie z.B. andere Ärzte) durch ihre Einrichtung angeboten?** (Mehrfachantworten möglich)	
☐ Angepasste Schulungen und Informationsveranstaltungen	☐ Gesprächsangebote (z.B. Einzelgespräche, Gesprächskreise) für unterschiedliche Religionsgruppen
☐ Möglichkeit, ausschließlich von Personen des eigenen Geschlechts behandelt zu werden	☐ Sonstiges, und zwar: ______________________
☐ Die genannten Angebote treffen bei unserer Einrichtungsart nicht zu	

**Welche der folgenden nicht-therapeutischen Angebote stehen in Ihrer Einrichtung zur Verfügung?** (Mehrfachantworten möglich)	
☐ Menüauswahl, die kulturelle Bedürfnisse berücksichtigt (z.B. koschere oder Helal-Kost)	☐ Möglichkeit, zu abweichenden Zeiten Essen zu erhalten (z.B. im Ramadan nach Sonnenuntergang)
☐ Betreuung/Begleitung durch kooperierende Geistliche verschiedener Religionen	☐ Sonstiges, und zwar: ______________________
**Bei stationären Einrichtungen:**	
☐ Übernachtungsmöglichkeiten für Lebenspartner/innen und/oder Familienmitglieder	☐ Möglichkeit, das eigene Zimmer kultur- bzw. religionssensibel zu gestalten
☐ Neutrales Abschiedszimmer	☐ Gebetsraum/neutral gestalteter Religionsraum
☐ Die genannten Angebote treffen bei unserer Einrichtungsart nicht zu	

**Wie werden die Wünsche von Patienten/innen im Hinblick auf ihre Versorgung durch Ihre Einrichtung erhoben?** (Mehrfachantworten möglich.)	
☐ Es findet keine Erhebung statt	☐ Mündlich während des Aufnahme-/Anamnesegesprächs
☐ Patienten/innen füllen einen Fragebogen aus	☐ Anders, und zwar: ______________________________

**Inwieweit werden folgende Informationen Ihrer Patienten/innen routinemäßig (z.B. im Rahmen der Anamnese) erhoben und ausgewertet (z.B. zum Zwecke des Qualitätsmanagements)?**	**Wird nicht erhoben**	**Wird erhoben**	**Wird erhoben und ausgewertet**
Muttersprache/Sprachkenntnisse (inkl. Deutschkenntnisse)	☐	☐	☐
Übersetzungsbedarf (bei nicht ausreichenden Deutschkenntnissen)	☐	☐	☐
Konfession	☐	☐	☐
Fähigkeiten zu lesen und zu schreiben	☐	☐	☐
Wertvorstellungen bzw. kulturspezifische Einstellungen (z.B. in Bezug auf Krankheit)	☐	☐	☐
Essgewohnheiten (z.B. vegetarische, koschere und Helal-Kost)	☐	☐	☐
Besondere Bedarfslagen (z. B. Bewegungseinschränkungen, Minderung des Seh- oder Hörvermögens)	☐	☐	☐
Sexuelle Identität (z.B. Transgender etc.)	☐	☐	☐
Familiäre Situation (z. B. minderjährige Kinder oder zu pflegende Angehörige)	☐	☐	☐
Sonstiges, und zwar: ______________________________	☐	☐	☐
Die ausgewerteten Daten werden dafür genutzt, um unsere therapeutischen und nicht-therapeutischen Angebote den Bedürfnissen der Patienten/innen anzupassen	☐ Ja	☐ Nein	☐ In Planung

Sonstige Aspekte der Diversitätssensibilität

**Wie wichtig ist Ihrer Meinung nach die Diversitätssensibilität einer Einrichtung für…**	**Vollkommen unwichtig**	**Eher unwichtig**	**Eher wichtig**	**Sehr wichtig**
…die Zufriedenheit von Patienten/innen	☐	☐	☐	☐
…die Zufriedenheit von Mitarbeiter/innen (z.B. durch bessere Behandlungs- und Prozessabläufe)	☐	☐	☐	☐
…den Behandlungsverlauf von Patienten/innen	☐	☐	☐	☐
…die Attraktivität der Einrichtung	☐	☐	☐	☐
Sonstiges, und zwar: ______________________________	☐	☐	☐	☐

**Bitte geben Sie an, inwieweit Sie den folgenden Aussagen zustimmen:**	**Stimme voll zu**	**Stimme eher zu**	**Stimme eher nicht zu**	**Stimme gar nicht zu**
Unsere Einrichtung geht auf die Bedürfnisse unterschiedlicher Patienten/innengruppen ein.	☐	☐	☐	☐
Es ist notwendig, unsere Einrichtung diversitätssensibel auszurichten.	☐	☐	☐	☐
Zukünftig ist eine stärkere Ausrichtung unserer Einrichtung auf diversitätssensible Aspekte vorgesehen.	☐	☐	☐	☐
Es ist wichtig, dass unsere Mitarbeiter/innen an Fortbildungen oder Schulungen zum Umgang mit Diversität teilnehmen.	☐	☐	☐	☐
Eine (stärkere) diversitätssensible Ausrichtung ist aufgrund der derzeitigen finanziellen Situation für unsere Einrichtung nicht realisierbar.	☐	☐	☐	☐
Eine diversitätssensible Ausrichtung wird für viele Einrichtungen zukünftig eine noch größere Rolle als heute spielen.	☐	☐	☐	☐
Praxisnahe Handlungsempfehlungen könnten die Implementierung von diversitätssensiblen Maßnahmen fördern.	☐	☐	☐	☐

**Welche Hindernisse sehen/sahen Sie bei der Umsetzung einer diversitätssensiblen Versorgung in Ihrer Einrichtung?** (Mehrfachantworten möglich)	
☐ Es gab/gibt keine Hindernisse	☐ Nicht zutreffend, da keine Absicht zur Umsetzung solcher Maßnahmen in unserer Einrichtung besteht
☐ Mangelnde Motivation zur Umsetzung solcher Maßnahmen seitens des Personals	☐ Nicht alle Entscheidungsträger/innen der Einrichtung sind von der Notwendigkeit solcher Maßnahmen überzeugt
☐ Organisatorische Schwierigkeiten	☐ Fehlende finanzielle Ressourcen
☐ Fehlende Anreize des Versorgungsträgers	☐ Unklarheit, wie solche Maßnahmen umgesetzt werden können
☐ Sonstiges, und zwar: ______________________________	

**Falls Sie weitere Anmerkungen zum Forschungsthema haben, dann notieren Sie diese gerne hier:**

Herzlichen Dank für Ihre Teilnahme!

Bitte schicken Sie den ausgefüllten Fragebogen im beiliegenden Umschlag bis zum 30.09.2020 direkt an die Universität Witten/Herdecke. Das Porto übernehmen selbstverständlich wir.
